# Intelligence in offspring born to women exposed to intimate partner violence: a population-based cohort study

**DOI:** 10.12688/wellcomeopenres.15270.1

**Published:** 2019-07-10

**Authors:** Kathryn M Abel, Hein Heuvelman, Dheeraj Rai, Nicholas J Timpson, Jane Sarginson, Rebekah Shallcross, Heather Mitchell, Holly Hope, Richard Emsley

**Affiliations:** 1 Centre for Women’s Mental Health, Manchester Academic Health Sciences Centre, Faculty of Biology, Medicine and Health Sciences, University of Manchester, Oxford Road, Manchester, M13 9PL, UK; 2 Greater Manchester Mental Health NHS Foundation Trust, Bury New Rd, Prestwich, Manchester, M25 3BL, UK; 3 Centre for Academic Mental Health, Population Health Sciences, Bristol Medical School, University of Bristol, Oakfield House, Oakfield Grove, Bristol, BS8 2BN, UK; 4 Avon & Wiltshire Mental Health Partnership NHS Trust, Jenner House, Langley Park, Chippenham, SN15 1GG, UK; 5 MRC Integrative Epidemiology Unit, Population Health Sciences, Bristol Medical School, University of Bristol, Oakfield House, Oakfield Grove, Bristol, BS8 2BN, UK; 6 School of Healthcare Science, Manchester Metropolitan University, John Dalton Building, Chester Street, Manchester, M1 5GD, UK; 7 Centre for Academic Primary Care, Population Health Sciences, Bristol Medical School, University of Bristol, Canynge Hall, 39 Whatley Road, Bristol, BS8 2PS, UK; 8 Biostatistics and Health Informatics Department, Institute of Psychiatry, Psychology and Neuroscience, King's College London, 16 De Crespigny Park, London, SE5 8AF, UK

**Keywords:** Intimate partner violence, population-based cohort, offspring IQ, ALSPAC

## Abstract

**Background: **Intimate partner violence (IPV) is a risk factor for developmental problems in offspring. Despite a high prevalence of IPV in the UK and elsewhere, the longer-term outcomes of offspring born to exposed mothers remain under-researched.

**Methods: **Population-based cohort study. We assessed IPV prevalence by type and timing for 3,153 mother-child pairs with complete data within our study population and examined associations between IPV and offspring IQ. We used multiple-imputation to evaluate bias due to our exclusion of observations with missing covariate data.

**Results: **Nearly one in five mothers reported IPV during the study period, with 17.6% reporting emotional violence and 6.8% reporting physical violence. Taking into account potential confounders, the IQ scores of children born to mothers exposed to physical violence remained lower than those of maternally unexposed children (full-scale IQ = −2.8 points [95%CI −4.9 to −0.7], verbal IQ = −2.2 [95%CI −4.4 to −0.1], performance IQ = −2.7 [95%CI  −5.0 to −0.5]) and odds of below-average intelligence (IQ<90) remained increased for full-scale (OR 1.48 [95%CI 1.03 to 2.14] and performance IQ (OR 1.48 [95%CI 1.08 to 2.04]) but not verbal IQ (OR 1.06 [95%CI 0.69 to 1.64]). Most physical violence occurred postnatally, and relative odds were most substantial when mothers were exposed to violence across pre-/perinatal and postnatal study periods (OR performance IQ<90 = 2.97 [95%CI 1.30 to 6.82]).

**Conclusions: **Maternal exposure to physical IPV is associated with lower offspring IQ at age 8. Associations persisted after adjusting for potential confounders and were driven by violence occurring postnatally.

## Introduction

The World Health Organisation (WHO) reports a global lifetime prevalence of intimate partner violence (IPV) among ever-partnered women of 30% in 2013, with a prevalence of 23% in high-income countries ^1^. In 2014, the Office for National Statistics (ONS) estimated that 24% of women aged 16 and over in England and Wales had, at some point, been exposed to violence by their intimate partners ^2^. Roughly 30% of IPV may start, or increase in severity, during pregnancy ^3, 4^. A recent study of 19 developed and developing countries reported prevalence rates of IPV during pregnancy of between 4% and 14% in some African and South or Central American countries and between 2% and 7% in two European countries and Australia ^5^.

In addition to the immediate harm to the mother, IPV during pregnancy is associated with health problems in offspring resulting from these pregnancies. Reports include medical problems in pregnancy and obstetric complications ^6– 12^, preterm birth ^6– 8, 10, 12^, fetal growth restriction ^6– 10, 12, 13^ and fetal or neonatal death ^6, 8, 9, 14, 15^. Studies of children maternally exposed to IPV during pregnancy suggest they are at greater risk of developmental, socio-emotional and behavioural problems in infancy ^16– 19^; characteristics that are associated with greater risk for later adverse life outcomes ^20, 21^.

IPV is more prevalent among younger women ^1, 5, 8, 22– 24^ of low socioeconomic status ^1, 22– 29^ and in families with alcohol or substance misuse ^1, 25, 30– 32^; characteristics which are also associated with the developmental outcomes of children born into these circumstances ^33– 41^. Furthermore, the mother's exposure to IPV puts her at greater risk of mental health difficulties ^42– 44^, which may affect mother-child interaction and introduce additional risk for the developing child ^16, 45, 46^.

Given the extent of violence to women, there is a striking lack of recent evidence linking IPV with child cognitive outcomes. Numerous studies highlight socio-emotional and behavioural problems in children exposed to IPV between their parents ^16– 19, 47– 49^, but few link IPV exposure to cognitive outcomes ^49^. This is of importance as associations between child cognitive problems and later-life disadvantages may be mitigated through timely identification and interventions for families experiencing IPV.

Evidence to date is limited to studies that were generally underpowered to stratify exposure by type or timing; or to examine key confounders like parental education, social class or income ^50, 51^. A recent study by Flach and colleagues ^16^ highlights the challenges of missing data in this field but does not examine child cognitive outcomes.

This study aimed to: 1) assess the prevalence and timing of reported IPV in a large British population-based sample, and 2) examine associations with offspring cognitive ability at age eight by type (emotional or physical) and timing (pre/perinatal or later postnatal) of IPV.

## Methods

### Study cohort

**Figure 1.**
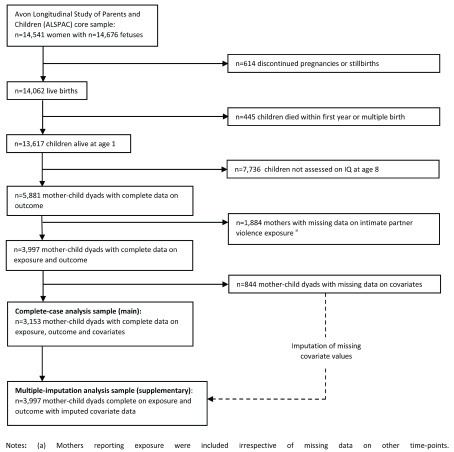
Selection of the study population.

The Avon Longitudinal Study of Parents and Children (ALSPAC) ^52^ is a population-based cohort of 14,000 pregnancies in three former Avon health districts. Women were recruited into the study between 1990 and 1992. All women pregnant with due-dates between April 1 ^st^ 1991 and December 31 ^st^ 1992 were eligible to take part. Prospective data collection began at 18 weeks gestation and included biological samples, questionnaire, interview and clinical data with the mother and child until the child reached adulthood. A fully searchable online data dictionary contains details of all available data ^53^. The cohort includes all mothers of single live births with complete exposure and outcome data. We describe the selection of our study population in Figure 1.

### Measures

***Intimate Partner Violence.*** ALSPAC routinely screens for violence within families through anonymised postal questionnaires completed by the mother. At 18 weeks’ gestation, mothers were asked if their partners had been emotionally or physically cruel to them since becoming pregnant and, at two months after birth of the study child, whether they had experienced emotional or physical cruelty by a partner since mid-pregnancy. At eight, 21, 33, 47, 61 and 73 months after birth of the study child, mothers were asked whether they had experienced emotional or physical cruelty by their partner since the prior questionnaire had been sent out. Response categories were: “ *Yes, and affected me a lot*”; “ *Yes, moderately affected*”; “ *Yes, mildly affected*”; “ *Yes, but did not affect me at all*”; and “ *No, did not happen*”.

To capture all IPV comprehensively, we considered a mother to be exposed if she reported cruelty, irrespective of the extent to which she reported having been affected by the experience. Furthermore, because the month two measure covered both pre- and perinatal time periods (i.e. from mid-pregnancy to two months after birth), we combined it with the measure at 18 weeks’ gestation and defined this as “pre- or perinatal exposure”, which covered the period from the start of pregnancy to two months after birth of the study child. Similarly, we combined all postnatal time-points into a single measure capturing postnatal exposure (i.e. from two months after birth until 73 months after birth).

We constructed the following exposure variables for use in analysis: (i) emotional IPV at any time from the start of pregnancy to 73 months postnatally; (ii) physical IPV at any time from the start of pregnancy to 73 months postnatally; (iii) emotional IPV during the pre- or perinatal period; (iv) emotional IPV during the postnatal period; (v) physical IPV during the pre- or perinatal period; and (vi) physical IPV during the postnatal period.

***Age eight cognitive ability.*** Childhood cognitive abilities at age eight were assessed using the Wechsler Intelligence Scale for Children (WISC) III test, which was administered by a member of the ALSPAC study team ^54^. This provided full-scale, verbal and performance IQ scores. We measured IQ as a continuous score and as a binary measure of below-average intelligence (IQ <90) ^54^.

***Pregnancy characteristics.*** We obtained measures for maternal age (in years), gestational age (term = 37 to 41 completed weeks, preterm = ≤36 completed weeks, and post-term = ≥42 completed weeks) and maternal use of alcohol (none versus any) or tobacco (none versus any) during pregnancy.

***Demographic and socioeconomic characteristics.*** Demographic characteristics included child sex and ethnicity (white versus non-white or mixed). Socioeconomic measures included the mother's and her partner’s social class (I/II: professional or managerial, III: skilled manual or non-manual, IV/V: semi-skilled or unskilled work) and educational attainment (degree, A-level, O-level or lower). In addition, we used a comprehensive measure of financial hardship experienced around birth of the study child, which included five questions on difficulties in providing shelter and sustenance for the study child, each rated from 0=not difficult to 3=very difficult. We added the scores for the individual questions to derive a summary measure and defined the following categories: no financial hardship (summary score = 0); moderate financial hardship (summary score = 1-7); and severe financial hardship (summary score = 8-15), where severe financial hardship represented the top decile of the summed scores.

***Maternal perinatal and postnatal depression.*** The Edinburgh Postnatal Depression Scale (EPDS) ^55^ was used to measure depressive symptoms at 18 and 32 weeks’ gestation and 33 months after birth of the study child. Mothers with EPDS scores ≥13 were considered at risk of clinical depression ^56, 57^. We used these variables to construct binary measures to indicate risk of prenatal depression (EPDS ≤12 versus ≥13 at 18 weeks or 32 weeks gestation) and risk of postnatal depression (EPDS ≤12 versus ≥13 at 33 months after birth of the study child). 

### Statistical analysis

Analyses were performed in Stata 14/MP ^58^. We examined the characteristics of the complete-case analysis sample using Pearson’s Chi-squared test to compare unexposed with exposed mother-child dyads. We then examined associations between the mother’s exposure to IPV and her offspring’s total, verbal and performance IQ at age 8, using linear regression models to examine associations with continuous IQ scores, and logistic models to examine odds of below-average intelligence associated with exposure. We adjusted our estimates to examine the influence of potential confounders in the following sequence: (1) unadjusted; (2) adjusted for pregnancy-related characteristics (offspring sex and ethnicity, gestational age at birth, maternal age, smoking and drinking during pregnancy); (3) adjusted for maternal depression (EPDS at 18 and 32 weeks’ gestation and 33 months after birth of the study child); (4) adjusted for socioeconomic characteristics (mother’s and partner’s social class and education and experience of financial hardship around birth of the study child); and (5) fully adjusted model (including all prior covariates). Additionally, we examined the odds of below average intelligence using a logistic regression model with statistical adjustment for all potential confounders (6). In the first set of models, we examined exposure to IPV by type of exposure (emotional versus physical) and in the second set by timing (only pre- or perinatal, only postnatal, exposed in both the pre- or perinatal and postnatal periods). To examine the influence of other violence within the family, we repeated our analyses where the mother reported that the child had not been exposed to violence by either parent.

In addition to the analysis of observations with complete data, we examined associations after imputing missing data for covariates using multiple imputation with chained equations (MICE) ^59^, as shown in Figure 1. We assumed covariate data were missing at random (MAR) conditional on the variables included in our analysis model and performed 100 imputations by 10 cycles of regression. We chose not to predict values for missing IPV exposures, as these data were deemed likely to be missing not at random (MNAR). We also did not predict values for missing offspring IQ scores as sufficiently predictive auxiliary data were not available. To explore potential selection bias, we assessed the characteristics of mother-child dyads with complete and missing data on IPV exposure status or IQ scores at age eight, using Pearson’s Chi-squared test to compare covariate distributions between groups.

The source code pertaining to the statistical analysis is provided (see *Software availability*) ^60^.

### Ethical statement

Ethical approval for the study was obtained from the ALSPAC Ethics and Law Committee and the Local Research Ethics Committee. Informed consent for the use of data collected via questionnaires and clinics was obtained from participants following the recommendations of the ALSPAC Ethics and Law Committee at the time. Full details of the approvals obtained and details regarding ethics approvals are available.

## Results

Characteristics of the complete-case analysis sample are described in Table 1. Nearly one in every five mothers (n=585, 18.6%) in our study population reported exposure to violence by an intimate partner from the start of pregnancy to 73 months after birth of the study child. Compared with unexposed mothers, exposed mothers were more likely to have non-white or mixed ethnic backgrounds, had an overall less favourable socioeconomic profile, were more likely to smoke or drink during pregnancy, were at greater risk of perinatal and postnatal depression and were more likely to have children with below-average IQ. The prevalence of exposure was moderately higher in the multiple-imputation analysis sample (n=817, 20.4%) although differences between exposed and unexposed mothers were consistent with those reported above (Table S1, see *Extended data*) ^61^.

**Table 1.** Characteristics of the complete-case analysis sample by the mother’s exposure status (n=3,153).

		Mother unexposed to IPV		Mother exposed to IPV		Pearson’s chi-square	
		N=2,568 (81.4%)		N=585 (18.6%)			
		%	n	%	n	χ ^2^	p
**Child is female**		49.5	1,270	49.7	291	0.02	0.90
**Child ethnicity**	White	**97.9**	2,513	**95.0**	556		
	Non-White or mixed	**2.1**	55	**5.0**	29	14.57	<0.001
**Gestational age at birth**	≤36 completed weeks	3.6	93	3.9	23		
	37-41 completed weeks	89.0	2,285	90.1	527		
	≥42 completed weeks	7.4	190	6.0	35	1.53	0.47
**Maternal age**	<20	0.4	11	0.9	5		
	20-35	90.5	2,325	88.7	519		
	>35	9.0	232	10.4	61	2.88	0.24
**Mother’s social class**	I / II	**45.7**	1,174	**39.0**	228		
	III non-manual / manual	**46.8**	1,202	**50.1**	293		
	IV / V	**7.5**	192	**10.9**	64	12.99	0.002
**Partner’s social class**	I / II	**55.9**	1,436	**43.8**	256		
	III non-manual / manual	**35.8**	918	**46.2**	270		
	IV / V	**8.3**	214	**10.1**	59	28.51	<0.001
**Mother’s education**	Degree	**50.0**	1,283	**44.3**	259		
	A-level	**35.1**	901	**38.0**	222		
	O-level or lower	**15.0**	384	**17.8**	104	6.70	0.035
**Partner’s education**	Degree	**57.7**	1,482	**49.7**	291		
	A-level	**21.3**	548	**20.9**	122		
	O-level or lower	**21.0**	538	**29.4**	172	20.54	<0.001
**Mother smoked during pregnancy**		**11.7**	301	**23.3**	136	53.02	<0.001
**Mother drank alcohol during pregnancy**		**12.4**	318	**17.3**	101	9.85	0.002
**Pre-/perinatal EPDS>13**		**14.2**	364	**35.7**	209	148.83	<0.001
**Postnatal EPDS>13**		**12.5**	321	**37.6**	220	211.29	<0.001
**Financial hardship**	None	**48.4**	1,244	**31.3**	183		
	Moderate	**46.1**	1,183	**54.9**	321		
	Severe	**5.5**	141	**13.9**	81	85.99	<0.001
**Child full-scale IQ <90 at age 8**		**13.0**	333	**16.8**	98	5.78	0.016
**Child verbal IQ <90 at age 8**		9.9	254	12.1	71	2.60	0.11
**Child performance IQ <90 at age 8**		**20.4**	523	**25.6**	150	7.90	0.005

Source: The Avon Longitudinal Study of Parents and Children (ALSPAC)

IPV, intimate partner violence; EDPS, Edinburgh Postnatal Depression Scale.

In the complete-case analysis sample, exposure to emotional violence was reported by 17.6% of mothers, while 6.8% of mothers reported exposure to physical violence ( Table 2). Accounting for a range of potential confounders, the adjusted IQ scores of children born to exposed mothers remained lower than those of children born to unexposed mothers (full-scale IQ = −2.8 points [95%CI −4.9 to −0.7], verbal IQ = −2.2 points [95%CI −4.4 to −0.1], performance IQ = −2.7 points [95%CI −5.0 to −0.5]) and odds of below-average intelligence remained increased for full-scale (OR 1.48 [95%CI 1.03 to 2.14]) and performance IQ scores (OR 1.48 [95%CI 1.08 to 2.04]) but not for verbal IQ scores (OR 1.06 [95%CI 0.69 to 1.64]). To our surprise, associations were moderately stronger when the mother reported that no violence had occurred between either parent and the study child (Table S2, see *Extended data*) ^61^ although it is possible that this result is biased by the under-reporting of parent-to-child violence for fear of intervention by social services, which may be more common in families where IPV is more severe. When we examined the multiple-imputation sample (Table S3, see *Extended data*) ^61^, the prevalence of IPV was moderately higher than in the complete-case analysis sample (emotional IPV=19.4%; physical IPV=8.3%), although we found moderately weaker residual associations between IPV and offspring IQ scores, suggesting that our complete-case analysis may have over-estimated the association between the mother’s exposure to IPV and offspring age eight IQ scores. Comparing the characteristics of dyads with complete and missing exposure or outcome data, those with missing data were more likely to have non-white or mixed ethnic backgrounds, children were born to younger mothers and more likely outside of term gestation, parents had an overall less favourable socioeconomic profile, mothers were more likely to smoke or drink during pregnancy, were at greater risk of perinatal and postnatal depression and were more likely to have children with below-average IQ (Table S4, see *Extended data*) ^61^. If these characteristics co-occurred with a greater prevalence of IPV (as suggested in Table 1), it is possible that our complete-case analysis estimate would have under-estimated the true association between IPV and offspring age eight IQ scores.

**Table 2.** Associations between maternal exposure to intimate partner violence (IPV) and offspring IQ in the complete-case analysis sample (N=3,153).

	Difference in offspring mean IQ score associated with exposure										Odds ratio for offspring IQ <90 associated with exposure		
	Model 1:		Model 2:		Model 3:		Model 4:		Model 5:		Model 6:		
	Crude estimate		Adjusted for pregnancy- related characteristics ^a^		Adjusted for maternal depression ^b^		Adjusted for socioeconomic characteristics ^c^		Fully adjusted estimate ^d^		Fully adjusted estimate ^d^		
	B ^e^	(95% CI) ^f^	B ^e^	(95% CI) ^f^	B ^e^	(95% CI) ^f^	B ^e^	(95% CI) ^f^	B ^e^	(95% CI) ^f^	n ^g^	OR	(95% CI) ^f^
**Exposure is any IPV ^h^** **Prevalence = 18.6% (n=585)**													
Outcome is offspring full-scale IQ	**-2.9**	**(-4.3 to -1.5)**	**-2.5**	**(-3.9 to -1.0)**	**-2.3**	**(-3.8 to -0.8)**	-1.1	(-2.4 to +0.2)	-1.0	(-2.4 to +0.4)	98	1.07	(0.82 to 1.41)
Outcome is offspring verbal IQ	**-2.7**	**(-4.2 to -1.2)**	**-2.3**	**(-3.8 to -0.8)**	**-2.0**	**(-3.6 to -0.5)**	-0.9	(-2.2 to +0.5)	-0.8	(-2.3 to +0.6)	71	0.98	(0.71 to 1.33)
Outcome is offspring performance IQ	**-2.4**	**(-3.9 to -0.9)**	**-2.0**	**(-3.5 to -0.5)**	**-2.1**	**(-3.6 to -0.5)**	-1.1	(-2.6 to +0.4)	-1.1	(-2.6 to +0.5)	150	1.14	(0.90 to 1.43)
**Exposure is emotional IPV** **Prevalence = 17.6% (n=555)**													
Outcome is offspring full-scale IQ	**-2.7**	**(-4.1 to -1.2)**	**-2.3**	**(-3.8 to -0.9)**	**-2.1**	**(-3.6 to -0.5)**	-0.8	(-2.2 to +0.6)	-0.8	(-2.2 to +0.7)	90	1.00	(0.76 to 1.33)
Outcome is offspring verbal IQ	**-2.6**	**(-4.1 to -1.1)**	**-2.3**	**(-3.8 to -0.7)**	**-1.9**	**(-3.5 to -0.3)**	-0.6	(-2.0 to +0.8)	-0.6	(-2.1 to +0.8)	69	1.01	(0.74 to 1.39)
Outcome is offspring performance IQ	**-2.2**	**(-3.7 to -0.6)**	**-1.9**	**(-3.4 to -0.3)**	**-1.8**	**(-3.4 to -0.2)**	-0.8	(-2.3 to +0.7)	-0.8	(-2.3 to +0.8)	139	1.08	(0.85 to 1.37)
**Exposure is physical IPV** **Prevalence = 6.8% (n=215)**													
Outcome is offspring full-scale IQ	**-5.6**	**(-7.8 to -3.4)**	**-4.9**	**(-7.1 to -2.7)**	**-4.9**	**(-7.1 to -2.7)**	**-3.0**	**(-5.1 to -1.0)**	**-2.8**	**(-4.9 to -0.7)**	49	**1.48**	**(1.03 to 2.14)**
Outcome is offspring verbal IQ	**-5.0**	**(-7.3 to -2.7)**	**-4.3**	**(-6.6 to -2.1)**	**-4.3**	**(-6.6 to -2.0)**	**-2.4**	**(-4.5 to -0.3)**	**-2.2**	**(-4.4 to -0.1)**	30	1.06	(0.69 to 1.64)
Outcome is offspring performance IQ	**-4.8**	**(-7.1 to -2.5)**	**-4.2**	**(-6.5 to -1.9)**	**-4.4**	**(-6.7 to -2.0)**	**-2.9**	**(-5.1 to -0.6)**	**-2.7**	**(-5.0 to -0.5)**	70	**1.48**	**(1.08 to 2.04)**

Source: The Avon Longitudinal Study of Parents and Children (ALSPAC).

Notes:(a) Pregnancy-related characteristics included child sex and ethnicity, gestational age at birth, maternal age, smoking during pregnancy and drinking during pregnancy. (b) maternal depression was defined as an Edinburgh Postnatal Depression Scale (EPDS) >13 at perinatal or postnatal time-points. (c) Maternal socioeconomic characteristics included mother’s and partner’s social class, mother’s and partner’s education and their experience of financial hardship around birth of the study child. (d) Association adjusted for all prior covariates. (e) B = the unstandardised regression coefficient which can be interpreted as the estimated difference in mean IQ score associated with exposure. (f) CI = confidence interval. (g) n = number of maternally exposed children with IQ<90. (h) Any IPV = emotional or physical IPV.

Given the lack of association between the mother’s exposure to emotional violence and offspring IQ after adjustment for confounding variables, we limited our analyses of timed exposures to those concerning physical violence ( Table 3). Most physical violence occurred between birth of the study child and 73 months after birth (n=169, 5.4%), while a smaller proportion of mothers were exposed solely during the pre- or perinatal period (n=20, 0.6%) or across prenatal and postnatal study periods (n=26, 0.8%). Following adjustment for potential confounding variables, the mother’s postnatal exposure to physical violence remained associated with lower offspring full-scale (−2.9 points [95%CI −5.2 to −0.6]), verbal (−2.4 points [95%CI −4.8 to 0.0]) and performance IQ scores (−2.7 points [95%CI −5.2 to −0.1]). Notwithstanding limitations in statistical power to examine these timed effects, exposure across both the prenatal and postnatal time periods was associated with near-threefold higher odds of below-average performance IQ in offspring (OR 2.97 [95%CI 1.30 to 6.82]). Associations were moderately weaker after the imputation of missing covariate data and postnatal exposure to physical violence appeared to be associated with offspring verbal rather than performance IQ scores in the multiple-imputation analysis sample (Table S5, see *Extended data*) ^61^.

**Table 3.** Associations between timing of mother’s exposure to physical intimate partner violence (IPV) and offspring IQ in the complete-case analysis sample (N=3,153).

	Mother exposed to physical IPV in the pre- or perinatal period only ^a^				Mother exposed to physical IPV in the postnatal period only ^b^				Mother exposed to physical IPV in both study periods			
	Prevalence = 0.6% (n=20)				Prevalence = 5.4% (n=169)				Prevalence = 0.8% (n=26)			
	Difference in offspring mean IQ associated with exposure		Odds ratio (OR) for offspring IQ <90 associated with exposure		Difference in offspring mean IQ associated with exposure		Odds ratio (OR) for offspring IQ <90 associated with exposure		Difference in offspring mean IQ associated with exposure		Odds ratio (OR) for offspring IQ <90 associated with exposure	
	Fully adjusted estimate ^c^		Fully adjusted estimate ^c^		Fully adjusted estimate ^c^		Fully adjusted estimate ^c^		Fully adjusted estimate ^c^		Fully adjusted estimate ^c^	
	B ^d^	(95% CI) ^e^	OR	(95% CI) ^e^	B ^d^	(95% CI) ^e^	OR	(95% CI) ^e^	B ^d^	(95% CI) ^e^	OR	(95% CI) ^e^
**Exposure is** **physical IPV**												
Outcome is offspring full- scale IQ	-0.9	(-7.4 to +5.5)	n/a ^f^		**-2.9**	**(-5.2 to -0.6)**	1.42	(0.94 to 2.13)	-3.6	(-9.3 to +2.1)	2.06	(0.85 to 4.97)
Outcome is offspring verbal IQ	+0.3	(-6.4 to +6.9)	n/a ^f^		-2.4	(-4.8 to 0.0)	1.13	(0.70 to 1.82)	-3.0	(-8.9 to +2.9)	n/a ^f^	
Outcome is offspring performance IQ	-2.4	(-9.4 to +4.7)	1.41	(0.52 to 3.87)	**-2.7**	**(-5.2 to -0.1)**	1.33	(0.93 to 1.91)	-3.4	(-9.7 to +2.8)	**2.97**	**(1.30 to 6.82)**

Source: The Avon Longitudinal Study of Parents and Children (ALSPAC)

Notes:(a) Exposure from conception to 2 months post-birth. (b) Exposure from birth to 6 years and 1 month post-birth. (c) Adjusted for child sex and ethnicity, gestational age at birth, maternal age, smoking during pregnancy, drinking during pregnancy, maternal Edinburgh Postnatal Depression Scale (EPDS) >13 at perinatal or postnatal time-points, mother’s and partner’s social class, mother’s and partner’s education and their experience of financial hardship around birth of the study child. (d) B = the unstandardised regression coefficient, which can be interpreted as the estimated difference in mean IQ score associated with exposure. (e) CI = confidence interval. (f) Number of maternally exposed children with IQ<90. (f) Odds ratio estimate not available due to cell count <5.

## Discussion

We examined associations between mothers’ exposure to IPV and offspring intelligence scores at age eight in a contemporary British cohort. There were three main findings: First, nearly one in every five women in our study population reported exposure to violence by an intimate partner. Emotional violence was reported by 17.6% of mothers and physical violence by 6.8% of mothers; although prevalence was higher when mother-child dyads with missing covariate data were included in the study sample after multiple-imputation (emotional violence=19.4%; physical violence=8.3%). Second, associations between the mother’s exposure to IPV and offspring intelligence scores at age eight were driven by her exposure to physical violence, with stronger associations for full-scale and performance IQ scores than for verbal IQ scores. Associations remained after statistical adjustment for a range of potential confounders, including detailed measures of socioeconomic position and financial hardship around birth of the study child. Third, most physical violence occurred after birth of the study child (n=169, 5.4%), although a small number of women reported only pre- or perinatal exposure (n=20, 0.6%) or exposure across the pre-/perinatal and postnatal study periods (n=26, 0.8%). Associations with offspring IQ appeared to be driven mainly by the mother’s postnatal exposure, although odds of offspring below-average intelligence were most substantial in women who were exposed to physical violence across both study periods. Contingent on the assumption of residually unconfounded effect, our findings suggest that there may be prenatal as well as postnatal mechanisms contributing to offspring intelligence scores at age eight, although our estimates were subject to low statistical power. Furthermore, if these mothers suffered more severe violence, it is possible that the effect associated with exposure in both study periods was strengthened by the severity of this exposure.

To our knowledge, this is the largest study of IPV to date reporting links to later offspring intelligence scores. We used multiple imputation to improve the accuracy of our estimates and to reduce the risk of selection bias because of missing covariate data. This study has several key strengths. It is the first study large enough to examine associations between IPV and child IQ by type and timing of exposure and with data to examine full-scale, verbal and performance IQ scores as separate outcomes. This is highly relevant because the extent of IPV means we must understand better which groups to target as a priority. High quality covariate measures allowed us to assess confounding by obstetric and parental socioeconomic characteristics and associations were investigated where the mother had reported that the child was not directly exposed to violence by either parent, thus isolating the potential effect of IPV from other violence within the family. Furthermore, use of anonymised postal questionnaires to record IPV has been shown to be preferable for women, as well as being effective in minimising the likelihood of missing data ^62^.

We also note several limitations. First, the measures used to capture IPV in ALSPAC did not include questions about sexual violence, nor ask for action-based examples of an intimate partner’s violent behaviour (such as those included in validated measures of IPV, e.g. the Composite Abuse Scale ^63^) which may lead to higher estimates of IPV prevalence ^16^. Second, women exposed to IPV may have been unable to complete questionnaires designed to capture these experiences for a variety of reasons. These may include presence of the intimate partner when the mother completed the written questionnaire, symptoms of post-traumatic stress as a result of prior violence, feelings of shame or stigma, or fear of intervention from services (for example, having children removed from the family). This would have resulted in exposure misclassification as well as, potentially, underestimation of IPV prevalence. Third, overlap in recall periods means that we were unable to distinguish clearly between exposure that occurred before or after birth. However, given that few women reported exposure solely in the pre- or perinatal period, this limitation may not have influenced our findings greatly. Fourth, we excluded children with missing IQ scores, as sufficiently predictive auxiliary data were not available. Attrition is common in cohort studies and known to be linked with socioeconomic factors which were controlled for in our analyses. Fifth, as in all studies employing non-randomised designs, there may have been residual confounding by unobserved variables. Specifically, intellectual disability in the mother (not available in our data), has been suggested, anecdotally, to be a risk factor for IPV ^64^ and may be an important residual confounder of associations between maternal exposure to IPV and offspring IQ scores, although the inclusion of maternal educational attainment in our models at least would have partly controlled for this.

Our findings extend earlier studies of IPV and child cognitive outcomes. Kitzmann and colleagues ^50^ performed a systematic review of smaller studies conducted between 1978 and 2000, finding below-average performance outcomes in 35 studies of academic achievement in children who had witnessed domestic violence. Similarly, Kolbo and colleagues ^51^ reviewed smaller studies conducted between 1975 and 1993, finding: below-average verbal and cognitive abilities; poor performance or failure in school; and increased risk of special educational needs in eight studies of cognitive outcomes in children witnessing IPV. Most of these earlier studies were conducted with clinical or service user samples, few controlled for socioeconomic confounding and there has generally been little acknowledgement of case-ascertainment or non-response bias in recruiting women exposed to domestic violence. Our study addresses these limitations, allowing for more robust conclusions to be drawn.

This study aimed to understand better the consequences of IPV for children. Overall, the current study demonstrates that postnatal exposure to physical IPV may have a negative effect on children’s IQ scores. Notably, the increase in the number of children falling within the below average range in those exposed to IPV, is of particular concern as cognitive disadvantages in childhood are associated with lower educational attainment in adolescence ^65, 66^ and may continue to affect individuals throughout their lives ^20, 21^. This area, therefore, warrants further investigation.

Our investigation of this contemporary British cohort suggests that IPV is common in the UK population today. Despite increasing awareness of this public health concern, the 2009 ‘Map of Gaps’ report ^67^ found a quarter of local authorities in Great Britain did not provide specialised services to women experiencing domestic violence. Our findings focus attention on children already vulnerable to the direct effects of violence exposure whose compromised cognitive ability may compound their risks across a broad range of life outcomes ^68^. This underlines the importance of early identification and intervention by services, not only for mothers experiencing IPV, who, in our sample, also show elevated rates of maternal depression, alcohol and cigarette consumption during pregnancy compared to unexposed mothers, but also for their children, if we are to mitigate potential long-term adverse effects ^69^.

The effects of IPV on child IQ that remain independent from socioeconomic and family factors are likely to include complex processes. One well-described corollary is dysregulation of the hypothalamic pituitary adrenal (HPA) axis in the context of compromised parental care, which has been linked to impaired neurocognitive and behavioural development ^70– 73^. These findings also accord with our previous reports detailing the postnatal effects of maternal severe psychological stress and increased risk of offspring neurodevelopmental disorder ^74, 75^.

Future work could be strengthened by the prospective monitoring of children following IPV, including measurement of specific cognitive abilities (i.e. memory, attention, processing speed, reasoning etc.) and by examining performance at pivotal moments (e.g. school reception and GCSE age) to elucidate the mechanisms by which deficits might occur and to quantify longer term effects of such exposures. Finally, further research is warranted to identify effective interventions to reduce exposure and to attenuate the impact of IPV for women and their offspring.

## Data availability

### Underlying data

The ALSPAC dataset contains personal and sensitive data; therefore, to make it freely available would be in contravention of the 2018 Data protection act. The data used in this paper can be made available through a system of managed open access. The following resources may be useful for navigating the application process: (i) The ALSPAC access policy ^76^ describes the process of accessing the data and samples in detail, and outlines the costs associated with doing so. (ii) A fully searchable research proposals database ^77^ lists all research projects that have been approved since April 2011. (iii) Access is granted to bona fide researchers who meet the requirements of the MRC Policy and Guidance on Sharing of Research Data from Population and Patient Studies. (iv) Proposals can be submitted online to the ALSPAC Executive Committee. You will receive a response within 10 working days to advise you whether your proposal has been approved ^77^.

### Extended data

Zenodo: Intelligence in offspring born to women exposed to intimate partner violence: a population-based cohort study. https://doi.org/10.5281/zenodo.3239226 ^61^

This project contains the following extended data:

- Extended data.docx (document containing Tables S1 – S5)

Data are available under the terms of the Creative Commons Attribution 4.0 International license (CC-BY 4.0).

## Software availability

**Source code available from:** https://github.com/HollyHope/IPV_IQ

**Archived source code at time of publication:** https://doi.org/10.5281/zenodo.3251881 ^60^

**License:** GNU General Public License v3.0
