# Supplementary material for: Intelligence in offspring born to women exposed to intimate partner violence: a population-based cohort study
Source: Wellcome Open Res. 2019 Jul 10;4:107. [Version 1] doi: 10.12688/wellcomeopenres.15270.1 (PMC6820818; doi:10.12688/wellcomeopenres.15270.1)
Supplement: Peer review history

# Peer review: Reviewer response for version 1

**DOI:** 10.21956/wellcomeopenres.16667.r36840

**Published:** 2019-10-29

**Authors:** Elisabete Pereira Silva

**Affiliations:** 1 Maternal and Child Department, Health Sciences Center, Hospital das Clínicas, Federal University of Pernambuco (UFPE), Recife, Brazil

The results of this study fill gaps in understanding the repercussions for cognitive development of children's exposure to maternal IPV during pregnancy and after birth.

 In my opinion, the study needs no modification. The abstract fulfils its role. The introduction provides an overview of the problem. The methods are described accurately. Statistical analysis was described and performed carefully. The problem of the attrition was properly controlled. The results and discussion were well elaborated and it shows the need for interventions to prevent exposure and mitigate the negative repercussions of IPV on women and their children.

I confirm that I have read this submission and believe that I have an appropriate level of expertise to confirm that it is of an acceptable scientific standard.

# Peer review: Reviewer response for version 1

**DOI:** 10.21956/wellcomeopenres.16667.r35959

**Published:** 2019-07-15

**Authors:** Myrna Weissman

**Affiliations:** 1 Department of Psychiatry, College of Physicians and Surgeons, Columbia University, New York, NY, USA

The ALSPAC again produces interesting and important results that require large samples, data on parents and children, and a longitudinal design. The finding is that partner violence during pregnancy and after is associated with decreased IQ in the child. This type of question in observational data requires a multitude of controls for confounders which can better explain the findings like e.g. maternal depression. The sample size allows for these analysis. This should get indexed without picking around the edges as the work is well done. Having said that the authors have an obligation I think to use their skills and this data set to try to understand the mechanism of this finding… is it maternal inattention, lack of play activities or schooling, too many other children, what is it that leads to this gross outcome lowered IQ? It is imperative that work be undertaken to understand and thus to intervene in these child victims. With maternal depression we could treat the mother, what can we do for these children other than stopping or preventing the violence? 

 As for the access to data the ALSPAC has a well worked up procedure.

I confirm that I have read this submission and believe that I have an appropriate level of expertise to confirm that it is of an acceptable scientific standard.

# Response

**Published:** 2019-07-15

**Authors:** Holly Hope

**Affiliations:** University of Manchester, UK

We thank the reviewer for their prompt and supportive review.

We appreciate that there remain important limitations to such an observational approach. As a result, we also agree that it is beholden on us to follow this work up to address potential mechanisms/further address confounding and we shall be applying for further funding in order to achieve this aim.
